# Biochemical and structural investigations on phosphoribosylpyrophosphate synthetase from *Mycobacterium smegmatis*

**DOI:** 10.1371/journal.pone.0175815

**Published:** 2017-04-18

**Authors:** Stefano Donini, Silvia Garavaglia, Davide M. Ferraris, Riccardo Miggiano, Shigetarou Mori, Keigo Shibayama, Menico Rizzi

**Affiliations:** 1 Department of Pharmaceutical Sciences, Università del Piemonte Orientale “A. Avogadro”, Largo Donegani 2, Novara, Italy; 2 Department of Bacteriology II, National Institute of Infectious Diseases, Gakuen 4-7-1, Musashimurayama-shi, Tokyo, Japan; Russian Academy of Medical Sciences, RUSSIAN FEDERATION

## Abstract

*Mycobacterium smegmatis* represents one model for studying the biology of its pathogenic relative *Mycobacterium tuberculosis*. The structural characterization of a *M*. *tuberculosis* ortholog protein can serve as a valid tool for the development of molecules active against the *M*. *tuberculosis* target. In this context, we report the biochemical and structural characterization of *M*. *smegmatis* phosphoribosylpyrophosphate synthetase (PrsA), the ortholog of *M*. *tuberculosis* PrsA, the unique enzyme responsible for the synthesis of phosphoribosylpyrophosphate (PRPP). PRPP is a key metabolite involved in several biosynthetic pathways including those for histidine, tryptophan, nucleotides and decaprenylphosphoryl-arabinose, an essential precursor for the mycobacterial cell wall biosynthesis. Since *M*. *tuberculosis* PrsA has been validated as a drug target for the development of antitubercular agents, the data presented here will add to the knowledge of the mycobacterial enzyme and could contribute to the development of *M*. *tuberculosis* PrsA inhibitors of potential pharmacological interest.

## Introduction

*M*. *tuberculosis* (MTB), the causative agent of tuberculosis (TB), is a pathogen as old as the human species that, even today, continues to be a threat for the entire world population. The World Health Organization Global Tuberculosis Report 2016 lists approximately 10.4 million new cases of TB worldwide, with about 1.4 million individuals succumbing to the disease [1]. Despite the availability of a number of effective first- and second-line drugs against TB, a rising threat is represented by the emergence of multi- and extensively drug-resistant MTB strains [2,3]. An additional complication for the treatment of TB is due to the ability of MTB to thrive in a dormant state in apparently healthy individuals, causing the asymptomatic infected carrier to be unaware of the disease, while retaining instead a pool of dormant bacteria in the body that can possibly resuscitate and that represent the reservoir of the disease. Such a dormant condition is overturned whenever an immunosuppressive condition occurs, as indeed observed in HIV-related immunodeficiencies, where dormant MTB bacteria are reactivated and spread, resulting in an active disease [4,5].

Anti-tubercular drugs targeting vulnerable targets and/or mechanisms of both replicating and non-replicating bacteria are critical for fighting TB. The biosynthesis of essential molecules such as nucleotides and amino acids represent attractive drug targets for fighting TB. In this context, phosphoribosylpyrophosphate (PRPP) is an essential molecule for the biosynthesis of purine and pyrimidine nucleotides, of NAD(P), as well as of the amino acids histidine and tryptophan [6,7]. The *M*. *tuberculosis* PrsA (MtPrsA) catalyzes the Mg^2+^-dependent conversion of ribose 5-phosphate (R5P) to PRPP using ATP, and is the solely enzyme responsible for PRPP synthesis in MTB [8]. Moreover, PRPP is required for the synthesis of arabinogalactan, and MtPrsA has been shown to be involved in the biosynthesis of decaprenylphosphoryl-arabinose, an essential precursors of cell wall components [9,10]. In addition, MtPrsA is upstream of DprE1 in this pathway [11], and DprE1 is a pre-clinically validated target with multiple inhibitors in development [12]. PrsA has been proved to be essential for *M*. *tuberculosis* survival and multiplication *in vitro*, and conditional knock-out mutant strains showed decreased viability and alteration in bacterial morphology [11]. Hence, MtPrsA is a critical enzyme for MTB metabolism and cell survival.

Three different classes of PrsA enzymes have been described based on the molecular and kinetic characterization. MtPrsA is classified as Class I PrsA due to its hexameric form, its substrate specificity for diphosphoryl donors, and the requirement of inorganic phosphate for enzyme activation [8,13,14]. MtPrsA shows approximately 40% sequence identity to the three PrsA human isoforms (hPrsA-1-3; >91% sequence identity between them) [8], and homology modelling revealed two major substitutions in the nucleoside triphosphate binding site, although the overall structure of the mycobacterial and human enzymes appears to be conserved [14].

*M*. *smegmatis* is a useful tool for studying *M*. *tuberculosis* in a laboratory setting due to its fastest doubling time, its non-pathogenicity in humans as well as its close genetic similarity to *M*. *tuberculosis* [15,16]. In this context, we report the structural and biochemical characterization of the PrsA enzyme from *M*. *smegmatis* (MsPrsA; 87% identity with MtPrsA), that is the first X-ray crystal structure of a mycobacterial PrsA ever determined. Our results demonstrate that the enzyme is a Class I PRPP synthetase and shows the typical structural architecture common to other PrsAs. The structural comparison between the MsPrsA and the human ortholog isoform 1, based on the optimal superimposition of the available experimentally determined crystal structures, highlighted few but significant differences in the enzyme active site that could be exploited for the design of specific MtPrsA inhibitors.

## Materials and methods

### Cloning

The gene encoding for MsPrsA was PCR amplified from *M*. *smegmatis* mc^2^155 (kindly provided by Prof. Giovanna Riccardi and Marilina Pasca, Department of Biology and Biotechnology 'Lazzaro Spallanzani', University of Pavia, Italy) using primers listed in Table 1. PCR-amplified DNA were cloned into a pCold™ I DNA vector (Takara Bio Inc.) by conventional methods. The constructed plasmids were transformed into *E*. *coli* DH5α (Invitrogen), and the integrity of the constructs was verified by restriction analysis, and inserted sequences were confirmed by DNA sequencing.

**Table 1:** Primer sequences (5’-3’) used in this study.

MsPrsA-Fw	gaacatatggccacggactggaccgac (underlined NdeI site)
MsPrsA-Re	gcctcgagttatgcagacccgtcgaacag (underlined XhoI site)

### Heterologous expression and purification

MsPrsA was expressed in Chaperon Competent *E*. *coli* BL21(DE3) Cell pTf16 (Takara Bio Inc.) in 2xTY medium in presence of Ampicillin (50 μg/ml) and Chloramphenicol (34 μg/ml). A single colony was inoculated into 20 ml of 2xTY supplemented with the same antibiotics and incubated overnight at 37°C. The culture was then split in two 5 L shaking flasks containing 1 L of 2xTY supplemented with the appropriate antibiotics. The bacterial cultures were incubated at 37°C until they reached an OD_600_ = 0.5, and the temperature was then lowered to 17°C. After 20 minutes incubation, isopropyl-1-thio-D-galactopyranoside (IPTG) was added to the bacterial culture at a final concentration of 0.5 mM following a 24-hours incubation at 17°C. After incubation, cells were harvested, and bacterial cell pellet was suspended in lysis buffer (Tris-HCl 50mM pH 8.0, NaCl 150mM, Glycerol 5%) containing protease inhibitor cocktail and Benzonase nuclease (Sigma-Aldrich) and mechanically lysed by sonication on ice. The lysate was centrifuged at 20000 rpm for 20 minutes, and the cleared cell lysate was loaded onto a Qiagen Ni-NTA Superflow column pre-equilibrated with lysis buffer. Column was thoroughly washed using ten column volumes of lysis buffer supplemented with 50mM imidazole, and the target enzyme was eluted with ten column volume of elution buffer (lysis buffer supplemented with 200mM imidazole). The fractions containing the target protein were pooled and then concentrated using an Amicon 20 with a molecular weight cut-off (MWCO) of 10000 Da. Concentrated fractions were loaded on a HiPrep 16/60 Sephacryl 200 High Resolution column pre-equilibrated with Lysis buffer. Fractions containing MsPrsA were concentrated using an Amicon 20 with 30000 MWCO.

All the purification steps described above were performed at 4°C. Concentrations were determined using the Bradford assay, and sample purity was assessed by 10% SDS-PAGE.

### Enzyme activity assay

PRPP synthase activity was assayed following a previously reported protocol [8]. The assay indirectly quantifies the amount of AMP produced as a product of the MsPrsA activity using an NADH-coupled enzyme system. The 200μl reaction mixture contained 50mM potassium phosphate buffer pH 7.5, 10mM Mg_2_Cl, 10mM KCl, 2mM ATP, 2mM R5P, 1.5mM PEP, 0.2mM NADH, 5U myokinase, 20U pyruvate kinase, and 10U lactate dehydrogenase (all the components were purchased from Sigma Aldrich). The enzyme activation by Mg^2+^ ion was tested at 0.5mM Mg-ATP, while activation by the inorganic phosphate ion (Pi) was measured using 50mM Tris HCl pH 7.5 and the curve was interpolated by the following equation (Eq 1):

$$v=\frac{V_{\max}}{1+\frac{K_{m}}{S}+\frac{S}{K_{i}}}$$ (1)

in which v, V_max_, S, and K_m_ represent, respectively, steady state reaction rate (μmol min^-1^ mg^-1^), maximum reaction rate, substrate concentration, and Michaelis-Menten constant for S. The enzymatic reaction was started by the addition of MsPrsA after 1 minute of incubation, and followed by the decrease in the absorbance at 340nm of NADH (6220 M^-1^ cm^-1^) at 25°C. One unit was defined as the amount of enzyme producing 1 μmol of NAD^+^ per minute, and specific activity was expressed as U mg^-1^ of enzyme. The assay was performed in quartz cuvettes using a Varian Cary 50-BiO UV-visible spectrophotometer equipped with a temperature-controlled cuvette holder.

### Substrate specificity and inhibition by purines ribonucleoside diphosphate

The diphosphate group (PPi) donor specificity was investigated using an enzyme-coupled continuous spectrophotometric assay as previously described [14] in which the MsPrsA reaction (R5P+ATP→PRPP+AMP) was coupled to *M*. *tuberculosis* orotate phosphoribosyl transferase forward reaction (PRPP+orotic acid→orotate mononucleotide phosphate + PPi; EC 2.4.2.10) and monitoring the decrease in absorbance at 295 nm caused by the conversion of orotic acid (OA ε_295_ = 3950 M^-1^ cm^-1^) in orotate mononucleotide phosphate (OMP). The diphosphate group donor specificity was investigated using guanosine triphosphate (GTP), cytosine triphosphate (CTP), and uridine triphosphate (UTP). The reaction was started by the addition of MsPrsA to the reaction mix after 1 minute of incubation. One unit is defined as the amount of enzyme necessary to convert 1 μmol of orotic acid in OMP per minute. Data points were obtained from three independent experiments. Pure recombinant MtOPRT production and purification are described elsewhere (Donini *et al*, *Scientific Reports*, in press).

In order to assess the inhibitory capacity of purines ribonucleoside diphosphate, inhibition assays were performed at fixed concentrations of R5P (60μM) and ATP (100μM) and varying the concentration of the ADP (0.2μM to 1mM) and GDP (0.02mM to 0.5mM), using the coupled assay described above. The concentration of inhibitor required to reduce the fractional enzyme activity to half of its initial value in the absence of inhibitor (IC_50_) by purine nucleoside diphosphate was calculated plotting the enzyme fractional activity against the logarithm of inhibitor concentration, and fitting the curves to a dose response curve using the following formula (Eq 2):

$$y=\min+\frac{\max-\min}{1+10^{\left(\mathrm{LogIC}50-x\right)}}$$ (2)

In the formula, y is the fractional activity of the enzyme in the presence of inhibitor at concentration [I], max is the maximum value of y observed at [I] = 0, and min is the minimum limiting value of y at high inhibitor concentrations.

### Steady state kinetics

The apparent steady-state parameters for MsPrsA over its natural substrates were determined using the NADH-coupled enzyme system. Substrates concentrations varied from 2 to 300μM and were of 2mM when held constant. Data points were obtained from three independent experiments.

Kinetic parameters were calculated using a non-linear least-square fit of the data using Sigma Plot Enzyme Kinetics Module 1.3 (Systat Software, San Jose, CA). ATP and R5P data were fitted, respectively, with Eq 3 (the Michaelis-Menten equation for hyperbolic substrate kinetics), and Eq 4 (the Hill equation for cooperative substrate saturation kinetics)

$$v=\frac{V_{\max}[S]}{K_{M}+[S]}$$ (3)

$$v=\frac{{V_{\max}[S]}^{n}}{{{(S}_{0.5})}^{n}+{\left[S\right]}^{n}}$$ (4)

in which v, V_max_, S, and K_m_ represent, respectively, steady state reaction rate (μmol min^-1^ mg^-1^), maximum reaction rate, substrate concentration, and Michaelis-Menten constant for S. S_0.5_ represents the half-saturation concentration for S, and n is the apparent Hill coefficient for S.

### Quaternary structure assessment

In order to assess the oligomeric state of the recombinant enzyme, the purified MsPrsA was dialyzed overnight against Tris-HCl 20mM pH 8.0, NaCl 50mM at 4°C, concentrated to a final volume of 1 ml (final MsPrsA concentration = 1mg/ml) and then subjected to an analytical gel filtration using a Sephacryl S200HR 16/60 High Resolution column (GE-Healthcare) equilibrated in the same buffer. For column calibration, the following proteins were used: Blue Dextrane (2000 kDa), catalase (232 kDa), aldolase (158 kDa), albumin (67 kDa), ovoalbumin (43 kDa).

### Multiple sequence alignment and protein-ligand interactions representations

The sequences alignment of PRPP synthetase from *M*. *smegmatis*, *M*. *tuberculosis* H37Rv, *B*. *subtilis*, and *H*. *sapiens* isoform 1 was performed using the T-Coffe Expresso online tool [17,18] and displayed by ESPript 3 [19]. Schematic protein-ligand representations were produced using LigPlot+ [20].

### Crystallization

Crystallization experiments were performed at 4°C by the sitting-drop vapour-diffusion method using commercially available screens (Qiagen, Hampton Research, Molecular Dimensions) and an Oryx4 Protein Crystallization Robot (Douglas Instruments Ltd.). Crystallization drops were prepared mixing 0.5 μl protein solution (5.5 mg/ml in 50 mM Tris HCl pH 8.0, 150mM NaCl) with 0.5 μl of reservoir solution (final mix pH = 7.4), and the droplet was equilibrated against 50 μl reservoir solution. Single, rectangular prism-shaped crystals grew after 90 days in a reservoir solution containing 0.2M NaCl, 0.1M Sodium acetate, 30% (v/v) 2-Methyl-2,4-pentanediol.

### X-ray diffraction and data collection

The MsPrsA crystal diffracted to 2.4 Å resolution on ID23-1 beamline at the ESRF in Grenoble (France). A full dataset was collected using a wavelength of 0.98 Å, a Pilatus 6M-F detector and a stream of liquid nitrogen at 100 K, without the use of cryoprotectant. Diffraction images were processed with XDS [21] and the crystal was assigned to space group P4_1_2_1_2 with unit cell parameters a = 85.2, Å b = 85.2 Å, c = 248.3 Å, α = β = γ = 90°. Data statistics are given in Table 2. The structure was determined by molecular replacement carried out with the program PHASER [22] of the PHENIX suite [23] and using the structure of a PRPP synthase from *B*. *subtilis* [24] (PDB: 1DKR) as the starting model. Model building was performed using AUTOBUILD [25], and refinement was carried out using REFMAC5 [26] and PHENIX [23]. The model was improved using Coot [27], and the stereochemistry of the structure was assessed using the program PROCHECK [28]. Figures were produced with PyMOL [29]. The coordinates and structure factors were deposited in the Protein Data Bank with accession code 5MP7.

**Table 2:** Data collection and refinement statistics.

Wavelength (Å)	0.98
Resolution range (Å)	43.38–2.4 (2.49–2.4)
Space group	P4_1_2_1_2
Unit cell (Å, °)	85.3, 85.3, 249.8, 90, 90, 90
Total reflections	68651 (6745)
Unique reflections	35723 (3511)
Multiplicity	1.9 (1.9)
Completeness (%)	96.18 (97.26)
Mean I/sigma(I)	6.24 (2.34)
Wilson B-factor (Å^2^)	38.07
R-merge	0.067 (0.34)
R-meas	0.094 (0.49)
Reflections used in refinement	35678 (3511)
Reflections used for R-free	1819 (159)
R-work	0.21 (0.29)
R-free	0.23 (0.31)
Number of non-hydrogen atoms	6944
macromolecules	6864
ligands	12
solvent	68
Protein residues	894
RMS(bonds)	0.014
RMS(angles)	1.68
Ramachandran favored (%)	97.85
Ramachandran allowed (%)	1.81
Ramachandran outliers (%)	0.34
Rotamer outliers (%)	2.75
Clashscore	6.45
Average B-factor (Å^2^)	42.98
macromolecules	43.04
ligands	40.17
solvent	38.25

Statistics for the highest-resolution shell are shown in parentheses.

### Docking studies

The Molecular Operating Environment (MOE; Chemical Computing Group Inc., Montreal, QB, Canada) was used for ligands docking simulation on the refined MsPrsA X-ray crystal structure, using the MOE LigX application with the Amber10: ETH force field under default parameters. The R5P binding site was detected using the MOE Alpha Site Finder application and by referring to the structure of *Thermoplasma volcanium* PRPP synthetase in complex with ribose-5-phosphate [30] (PDB code: 3MBI; 29% identity with MsPrsA). The model of the MsPrsA in complex with ribose-5-phosphate was thus determined using the MOE Dock application with the induced fit methods, which allows the receptor to move during refinement, under default parameters. Finally, the predicted structure was energy minimized using the default parameters of the MOE energy minimization algorithm (gradient: 0.10, Force Field: Amber 10).

As the same procedure adopted above failed to provide an unambiguous ADP binding mode, we modelled ADP-Mg^2+^ based on the optimal structural superimposition of our MsPrsA onto the crystal structure of PrsA from *T*. *volcanium* [30] in complex with ADP-Mg^2+^ (PDB code: 3MBI). The two structures could be superimposed with a root mean square deviation (r.m.s.d.) of 1.86 Å based on 238 Cα pairs. The resulting MsPrsA- ADP-Mg^2+^ binary complex was subjected to energy minimization in the same manner as described above for the complex with R5P.

## Results

### Expression and purification

**Fig 1:**
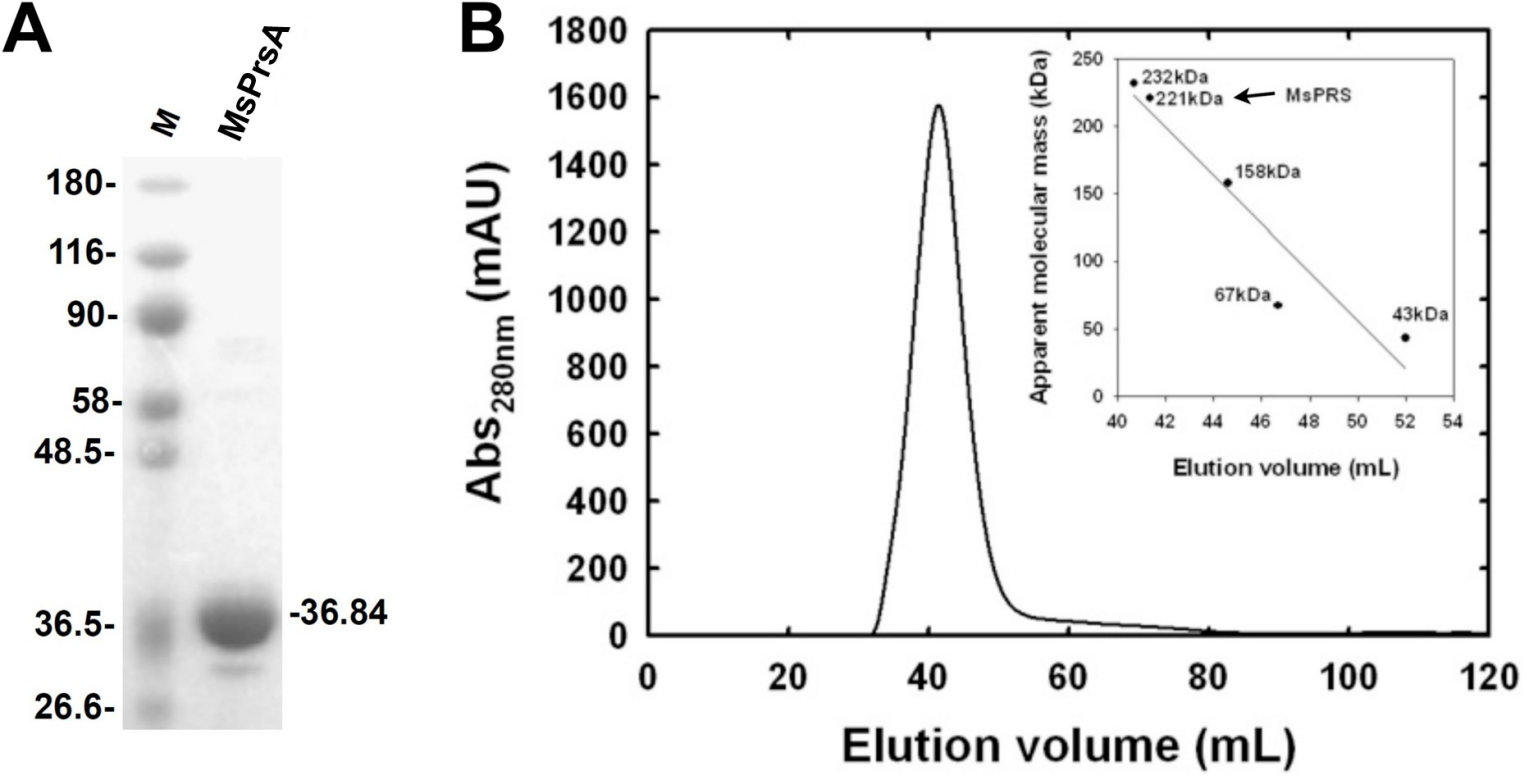
MsPrsA quaternary structure assessment. (A) 10% SDS-PAGE of pure recombinant MsPrsA: molecular weight markers are on the left (M) and the pure enzyme on the right showing a molecular weight of about 37 KDa. (B) Elution profile of MsPrsA on a HiPrep 16/60 Sephacryl 200 High Resolution pre-packed column. In the inset, the arrow indicates the MsPrsA elution volume on the experimentally determined calibration curve.

The expression and purification of the recombinant MsPrsA resulted in a pure and active protein with a yield of 4.5 mg of enzyme per liter of bacterial culture (Table 3). Noteworthy, compared to its ortholog in *M*. *tuberculosis* [8,14], the recombinant MsPrsA did not show any aggregate or any different oligomerization form than an hexameric quaternary structure typical of the Class I phosphoribosylpyrophosphate synthase [31,32], as shown by the lack of peaks corresponding to the column exclusion volume, and by the eluition volume of the peak corresponding to the eluted protein (Fig 1).

**Table 3:** Protein yield from 2 L culture.

Operation Step	Total Protein (mg)	Total Activity (U)	Specific Activity (U/mg)	Fold Purification	Yield (%)
Crude Lysate	226.0	723.2	3.2	1	100
Ni-NTA	20.0	7.2	0.4	0.11	0.99
S-200	12.8	17.8	1.4	0.44	2.47
Final MsPrsA	9.1	12.7	1.4	0.44	1.76

### Biochemical characterization

**Fig 2:**
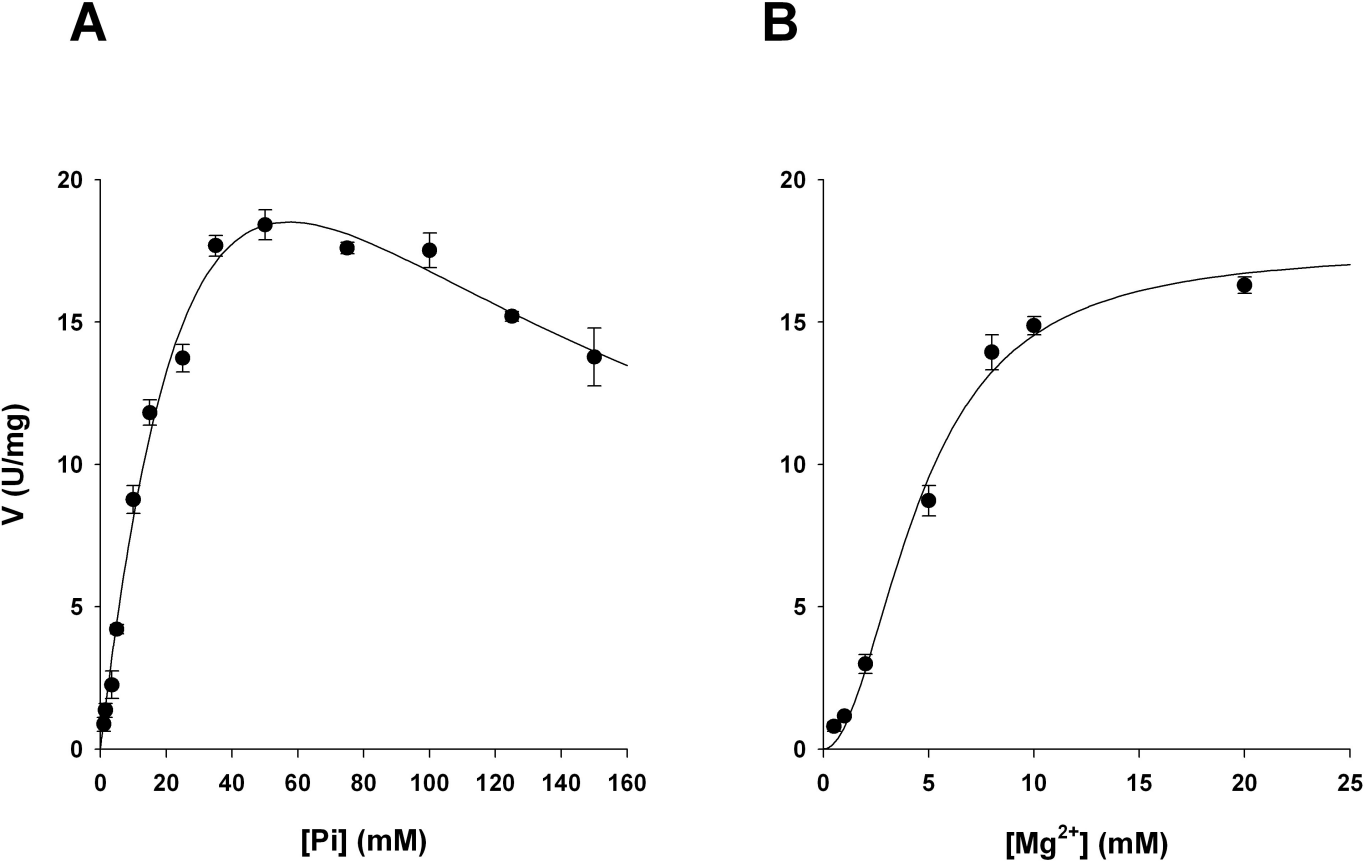
MsPrsA activation by Pi and Mg^2+^ ions. The enzyme activity was measured based on the NADH-coupled enzymatic assay described in Materials and Methods. (A) The MsPrsA activity was measured at pH 8.0 Tris HCl 50mM, varying the concentration of potassium phosphate at pH 7.5, between 1 to 150mM. (B) The enzyme activity was measured at pH 8.0 Tris HCl 50mM, varying the magnesium concentration from 0.5 to 20mM, at 5 mM R5P and 0.5 mM Mg-ATP fixed concentrations. Results are the mean values of three replicates.

We expected MsPrsA to belong to the Class I phosphoribosylpyrophosphate synthase subfamily, as previously demonstrated for the strict ortholog *M*. *tuberculosis* enzyme [8,13].Indeed, as expected for a Class I PrsA [33,34], we show that the *M*. *smegmatis* enzyme requires Pi with an optimal concentration ranging from 30 mM to 50 mM, with maximal activity peaking at 50 mM (Fig 2A). Noteworthy, like its *M*. *tuberculosis* ortholog, the *M*. *smegmatis* enzyme resulted to be allosterically activated by Mg^2+^ (S_0.5_ = 4mM, n = 2) (Fig 2B) as evidenced by the sigmoid curve of the catalytic rate plot at low concentration of Mg^2+^.

**Fig 3:**
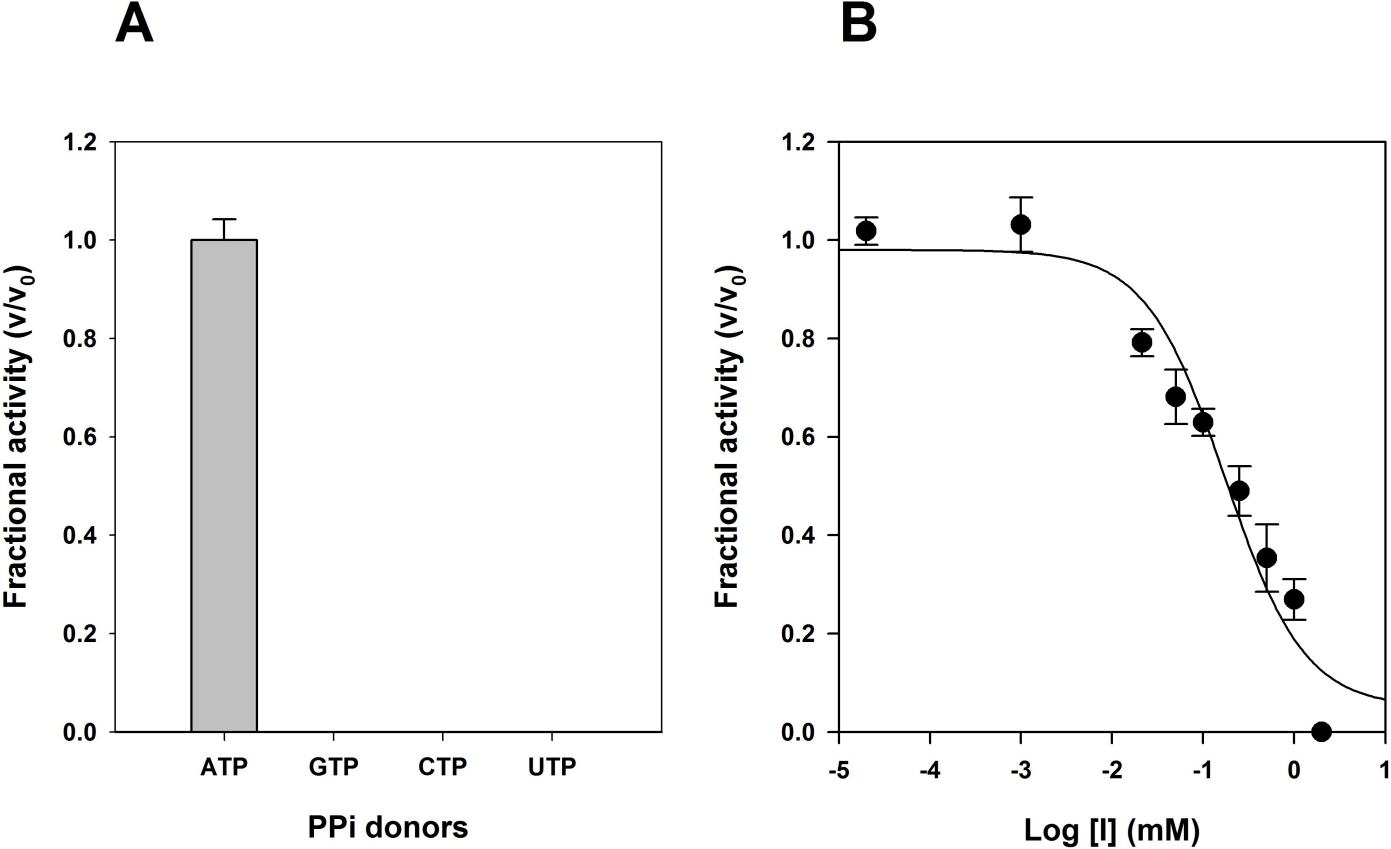
Substrate specificity for different PPi donors and inhibition by purines ribonucleoside diphosphate. The enzyme activity was determined by using a continuous spectrophotometric coupled assay as described in Materials and Methods. (A) The MsPrsA activity was measured at a concentration of 60μM for R5P, and of 100μM for each PPi donors tested. (B) Inhibition curve for ADP. The MsPrsA activity was measured at a fixed concentration of 60 μM for R5P and 100 μM of ATP, and varying the concentration of ADP (0.2μM-1mM); The data represent the average of three independent experiments.

Since Class I PRPP synthase are selective for ATP as a diphosphate donor, we investigated the specificity of the *M*. *smegmatis* enzyme toward ATP for its catalytic activity. The diphosphate donors GTP, CTP and UTP are not processed by MsPrsA, as expected for Class I PRPP synthases (Fig 3A). All Class I PrsA are reported to be inhibited by purines ribonucleotide diphosphate [8,13,33], and we therefore investigated whether ADP and GDP inhibits the *M*. *smegmatis* enzyme. Data analysis showed that ADP inhibited MsPrsA activity with a calculated IC_50_ of 174 ± 46 μM (Fig 3B), while GDP acted as a partial inhibitor, retaining about 50% of MsPrsA enzymatic activity even at concentration of 5 mM (data not shown).

**Fig 4:**
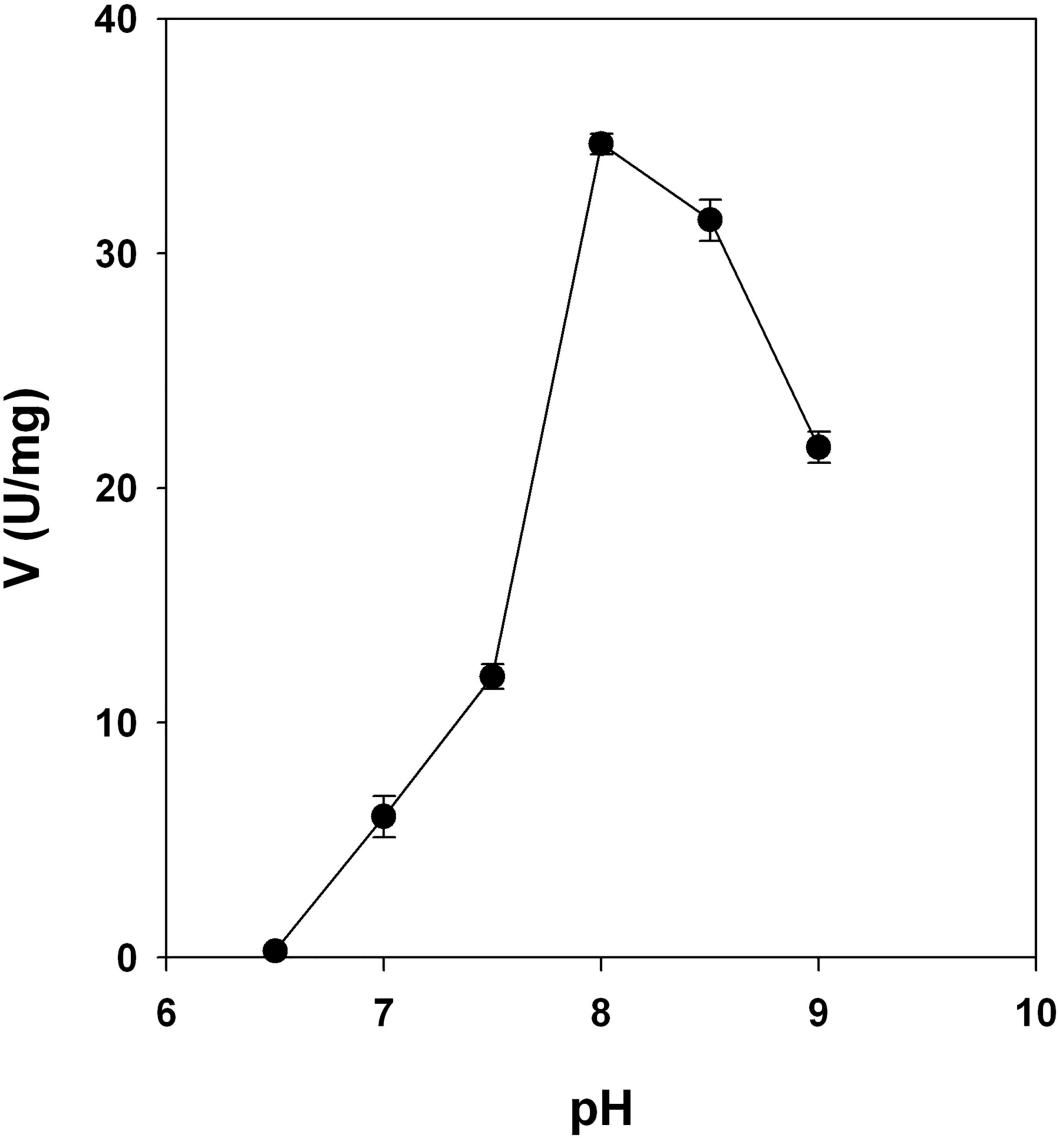
pH-activity profile for MsPrsA. The enzyme activity was determined by using a continuous spectrophotometric coupled assay as described in Materials and Methods. The effect of pH on the activity of MsPrsA was determined at 5 mM R5P, 5 mM ATP and 50 mM Pi, using the following buffers: KPi (50mM, pH range 6.5–8), Tris-HCl (50mM, pH range 8.5–9). Results are the mean values of three replicates.

We measured also the PrsA enzymatic activity at different pHs (Fig 4), and the catalytic activity reached a peak at a pH close to 8, retaining 50% of activity at pH = 9, and showing a pH activity profile similar to the one observed for MtPrsA [13].

The quaternary structure featuring PRPP synthetase was reported to be essential for catalysis [35–37], and we therefore investigated the oligomeric state of *M*. *smegmatis* PrsA through gel filtration chromatography. The oligomeric state for the recombinant, pure MsPrsA is consistent with an hexameric quaternary structure in solution as calculated from the elution volume that is consistent with a molecular weight of about 221 KDa (Fig 1).

**Fig 5:**
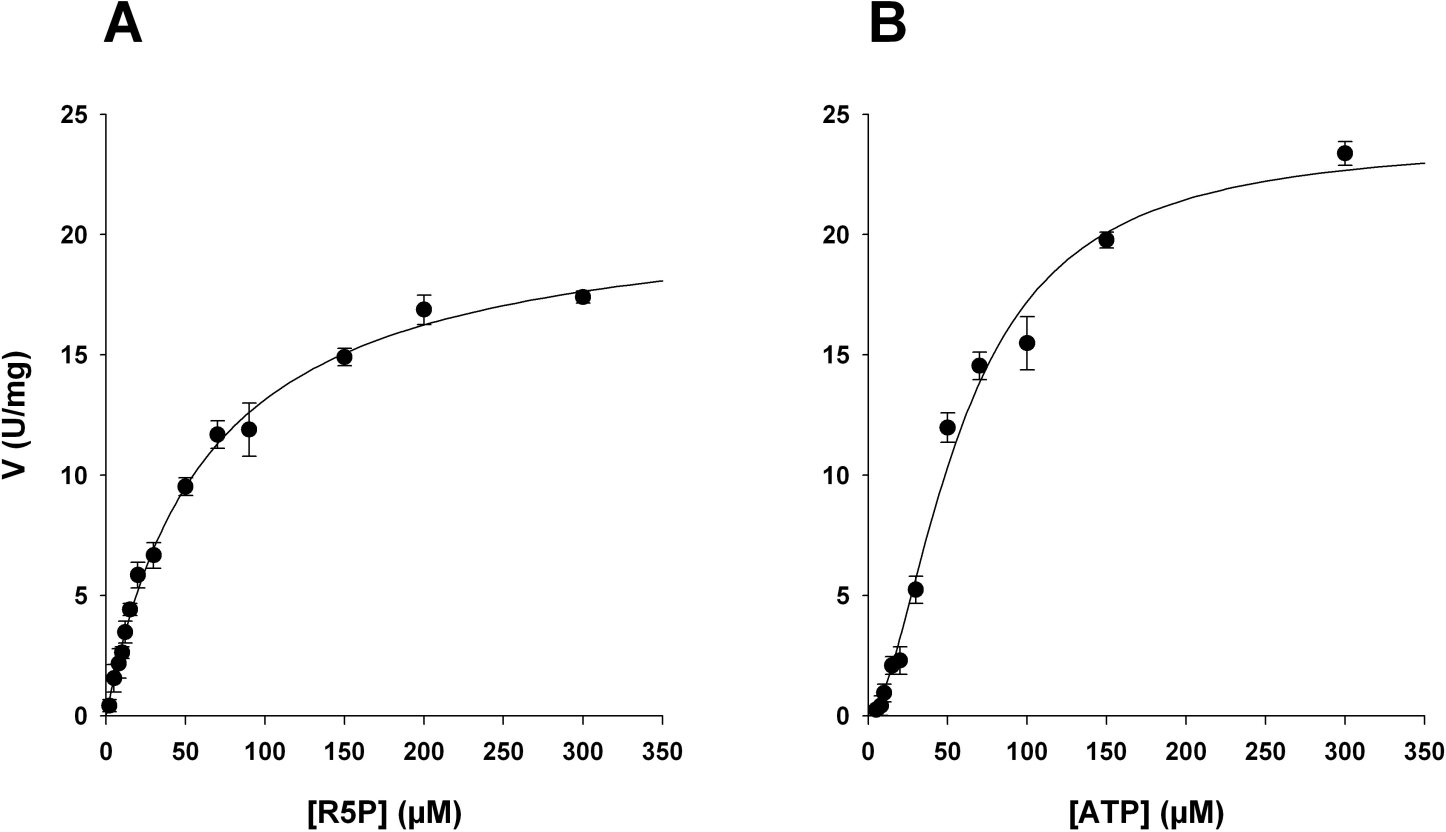
MsPrsA catalytic activity for R5P and ATP. The MsPrsA activity was measured at pH = 8.0 in Tris HCl 50mM, varying the concentration of R5P and ATP. Results are the mean values of three replicates.

The MsPrsA catalytic parameters for R5P and ATP were calculated. As for all Class I PRPP synthases, the calculated initial velocity (v_0_) as a function of the R5P concentration resulted in a hyperbolic curve typical of Michaelis-Menten kinetics (Eq 1), while a sigmoidal curve was observed with ATP, indicating positive co-operativity for this substrate as typically reported for a Class I PRPP synthetase (Fig 5). Indeed, the sigmoid curve was well fitted by the Hill equation (Eq 2), with an apparent Hill coefficient (n) of 1.7. All the kinetic data are summarized in Table 4 and are in line with the kinetic parameters measured at saturating concentration of Pi (50mM) and previously reported for MtPrsA [8].

**Table 4:** Kinetics parameters of MsPrsA in presence of Pi 50mM.

	V_max_ (μmol s^-1^)	k_cat_ (s^-1^)	K_m_/S_0.5_ (mM)	K_cat_/(K_m_/S_0.5_) (s^-1^ mM^-1^)	Hill coeff.(n)
R5P	9.4x10^-5^ ± 3x10^-6^	77.6 ± 2.5	0.059 ± 0.005	1315.2 ± 69.6	-
ATP	10x10^-5^ ± 5x10^-6^	83.5 ± 4.1	0.058 ± 0.005	1439.6 ± 53.8	1.7 ± 0.15

### Overall structure

**Fig 6:**
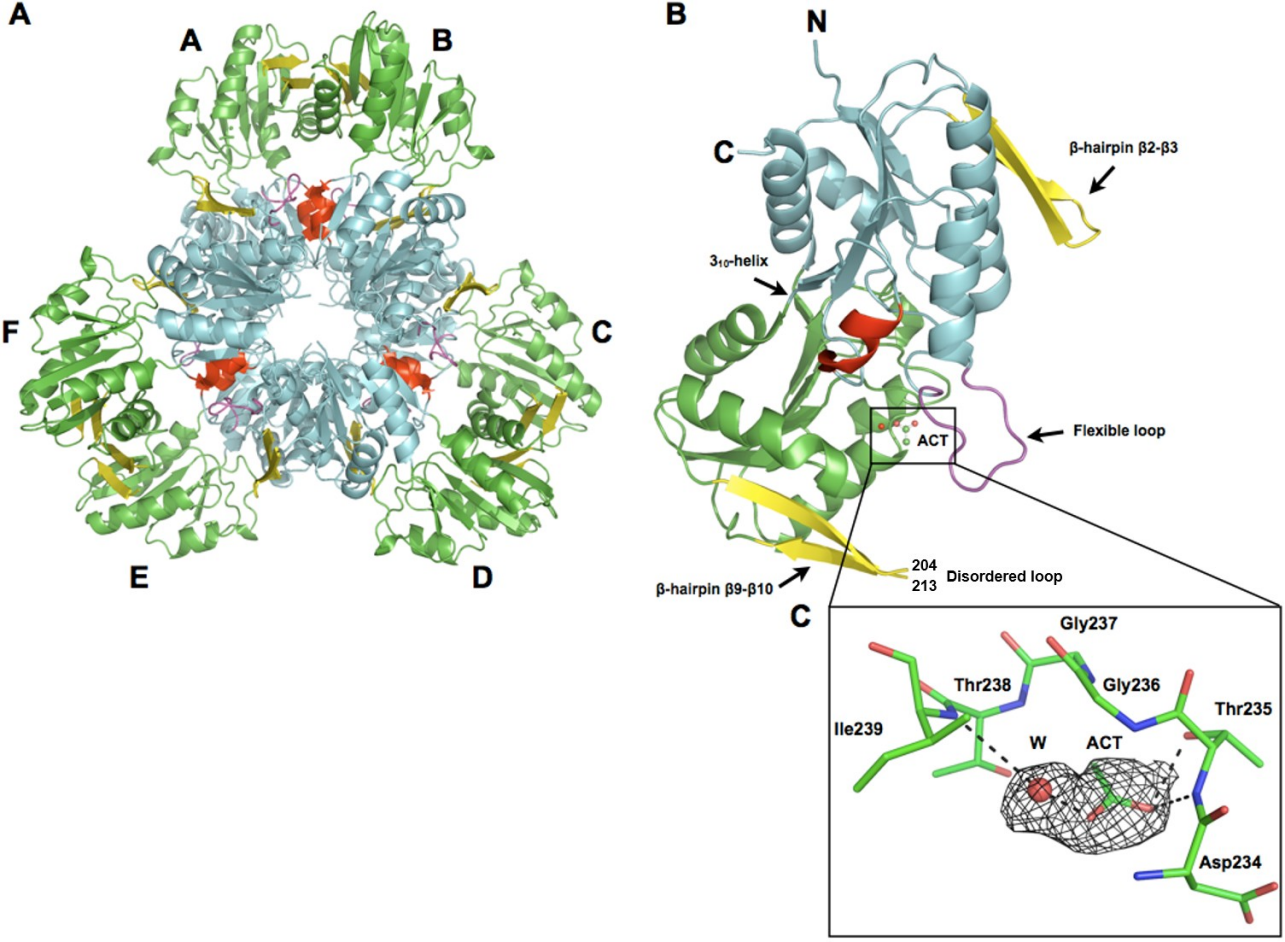
The overall structure of MsPrsA. Ribbon representation of the quaternary structure consisting of an hexameric arrangement (A) and of the monomer fold (B). Domains are colored in cyan (N-terminal) and green (C-terminal). The 3_10_-helix and the flexible loop, part of the N-terminal domain, are colored in red and magenta, respectively. Flag regions (β-hairpin β2-β3, and β9-β10) are shown in yellow, and the two extremities of the disordered loop are indicated (residues 204 and 213). The acetate molecule (ACT) is shown as ball and stick and the water is represented as a red sphere. (C) Magnified view of R5P binding loop with protein residues and acetate shown as ball-and-stick and the water molecule as a red sphere; relevant interactions are indicated with black dashes. The 2Fo-Fc electron-density map has been contoured at 1.0σ level.

The final MsPrsA structure contains residues from Arg9 to Gly316, with no electron density observed for the first eight amino-acids and for the stretch Arg204-Lys213. A Matthews parameter [38] and a solvent fraction of 2.11 Å Da^-1^ and 41.76%, respectively, were calculated indicating the presence of three protein subunits in the asymmetric unit. The physiological hexamer was generated by applying a two-fold crystallographic symmetry operators to the trimer. The resulting hexamer is endowed with a 32-point symmetry with a two-fold axis perpendicular to a three-fold axis and shows the subunits arranged in a propeller-shaped cyclic assembly with the N-terminal domains oriented toward the inner side of the hexamer, close to the three-fold axis (Fig 6A).

Each monomer shows the typical structural organization previously described in several PRPP synthetases [24,30,31,39], with two almost identical domains related by a pseudo two-fold symmetry (Fig 6B). Each domain consists of an ɑ/β fold built on a central five-stranded parallel β-sheet surrounded by four ɑ-helices with the N-terminal domain (residues Thr139-Phe146) containing an additional 3_10_ helix. The flexible loop encompassing residues Gln105 to Ile115 bridges the two domains (Fig 6B). As described for other PRPP synthetases, both domains contain a β-hairpin flag region that protrudes from the central core. In the N-terminal domain this region is composed by the loop joining the β2-β3 strands (residues Ala37-Asp53) and is part of the active site in the opposing subunit of a functional dimer, while in the C-terminal domain this region consists of the loop joining the β9-β10 strands (residues Pro196-Val218). This region is involved in the conformational changes occurring at the active site, and plays a critical role in catalysis. In all the subunits of MsPrsA one molecule of acetate (ACT) sits in the phosphate binding pocket of the ribose-5-phosphate binding site and makes specific contacts with residues defining the R5P binding loop (amino-acids Asp230-Thr238) (Fig 6C).

### Structural comparison with human PrsA-I

**Fig 7:**
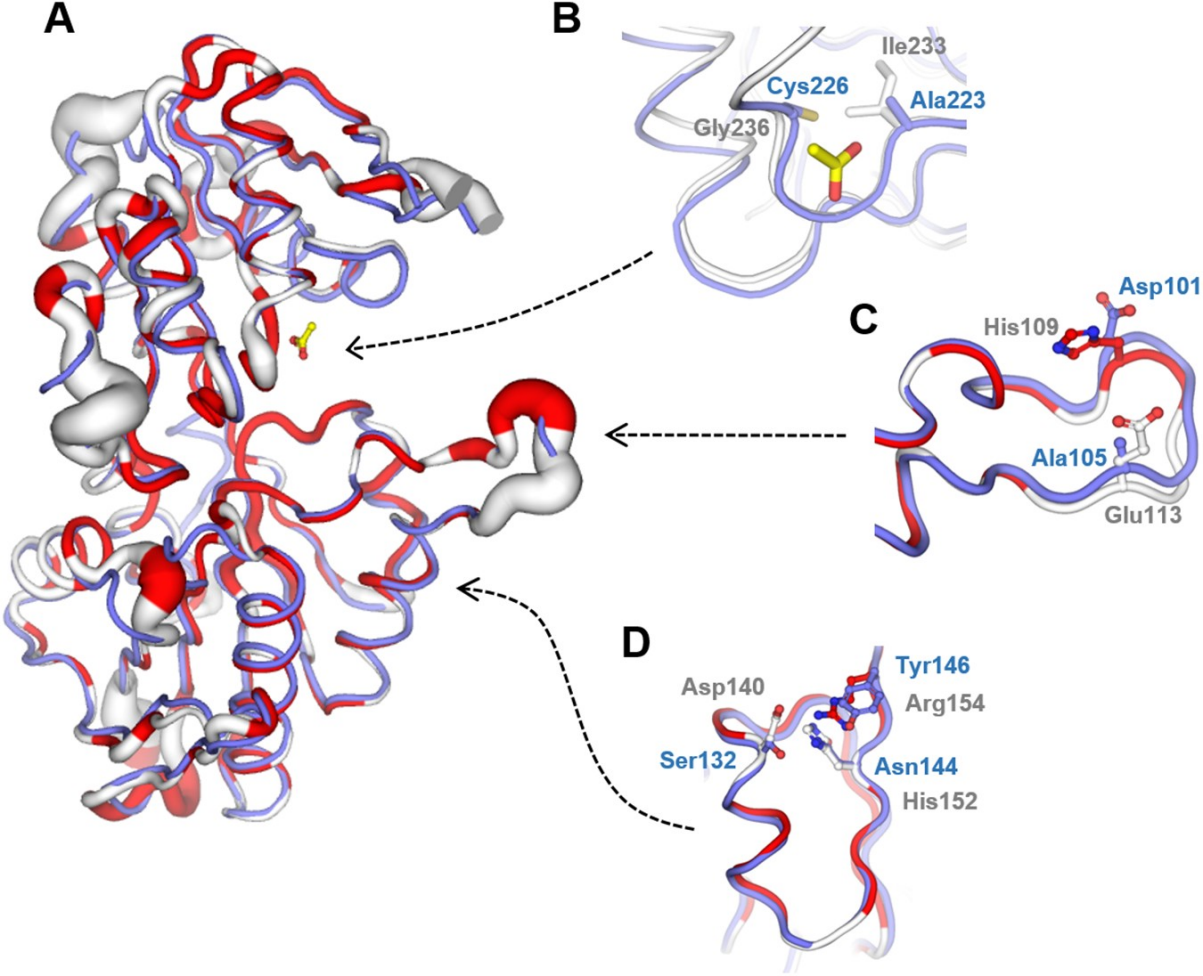
Structural comparison of human and *M*. *smegmatis* PrsA. (A) Superimposed structures with hPrsA-I (PDB: 2HCR) shown in light blue and MsPrsA with a white-red colour and sausage representation scheme according to the degree of amino-acids sequence conservation defined as follows: red, conserved; white, non-conserved; sausage radius proportional to the sequence alignment score: the smaller the radius the higher the conservation. Close-up views of the R5P binding site (B), the flexible loop part of the ATP binding site (C) and the allosteric regulatory site (D). Same color scheme as above and sausage representation omitted for clarity.

**Fig 8:**
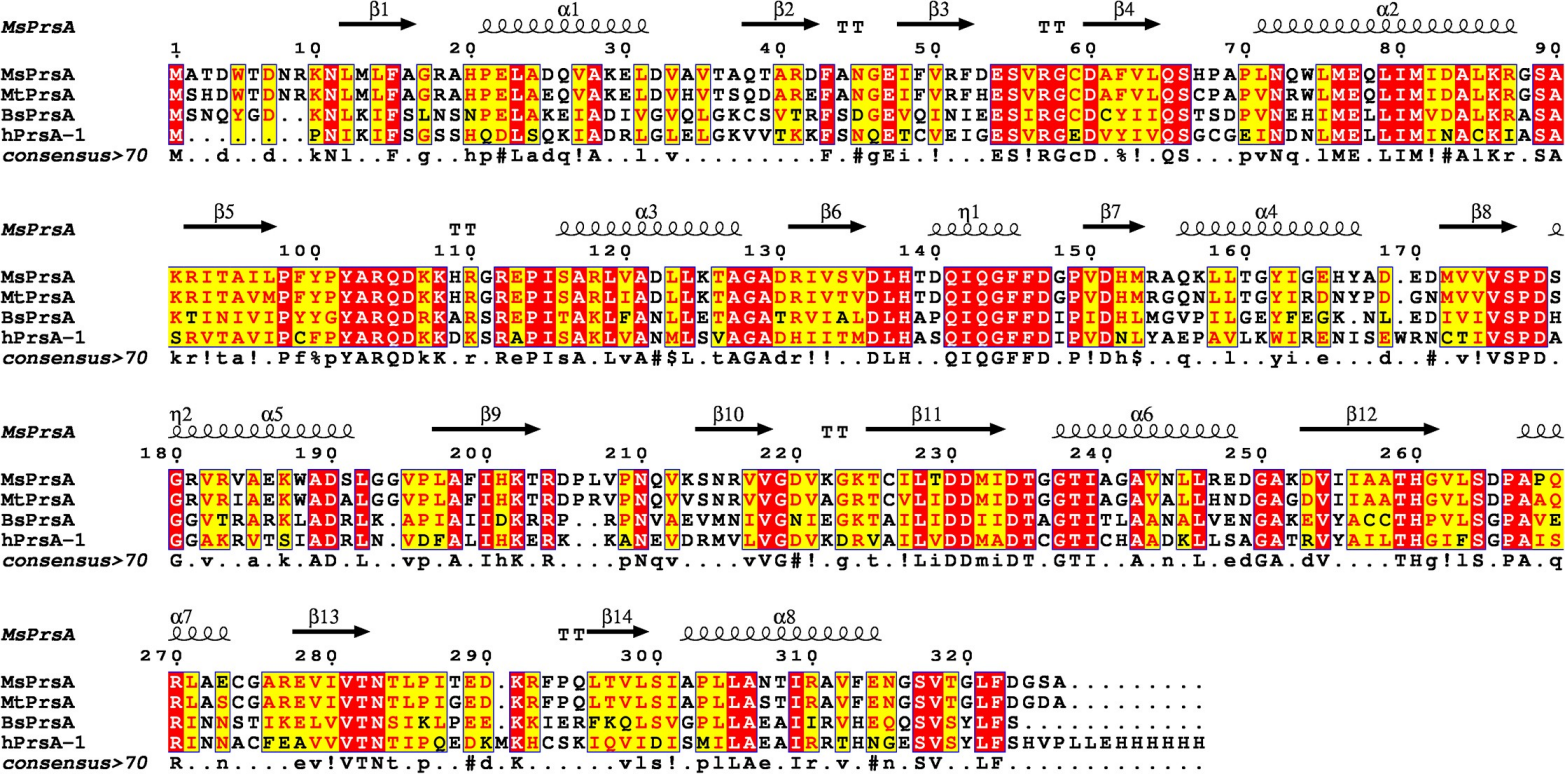
Sequence alignment of PrsA from *Mycobacterium smegmatis* (MsPrsA), *Mycobacterium tuberculosis* H37Rv (MtPrsA) *Bacillus subtilis* (BsPrsA), and human isoform 1 (hPrsA-1). Conserved residues are shown on a red background whereas chemically similar residues on a yellow background. The sequence identities between MsPrsA, MtPrsA, BsPrsA, and hPrsA are 88%, 46%, 43% respectively. Numbering and secondary structure elements assignments are shown for the mycobacterial enzyme. Consensus sequence information are edited according to the Espript3 [19] notation conservation.

The structures of *M*. *smegmatis* PrsA and hPrsA-1 (PDB code: 2HCR) were superimposed with a r.m.s.d of 0.64 Å based on 252 Cα pairs. Although the structural comparison and sequence analysis with the human enzyme revealed a strong conservation (Figs 7A and 8), few significant differences in the three different sites critical for the enzyme activity can be identified. In MsPrsA one molecule of ACT occupies the phosphate binding pocket of the ribose-5-phosphate binding site where a sulfate anion was observed in the structure of hPrsA-I [39]. Although the sequence identity between the mycobacterial and human enzymes in the region defining the R5P is very high, two differences emerge, with MsPrsA Ile233 and Gly236 occupying the structurally equivalent positions of Ala222 and Cys226, respectively, of the human enzyme (Fig 7B). As far as the ATP binding site is concerned, we observed a highly structural conservation between MsPrsA and hPrsA-I. However, also in this case, two significant substitutions are observed with MsPrsA His109 and Glu113 occupying the structurally equivalent position of Asp101 and Ala105 in the human enzyme, respectively (Fig 6C). Finally, the residues forming the allosteric site described in hPrsA-1 [39], that are strictly conserved in eukaryotic PrsAs, are not conserved in the mycobacterial enzyme. Indeed, Asp140, His152 and Arg154 of the MsPrsA replace Ser132, Asn144 and Tyr146, respectively, in hPrsA-I (Fig 7D). Considering that the all the key amino-acids in the actives sites of *M*. *tuberculosis* and *M*. *smegmatis* PrsA are strictly conserved, the differences with the human enzyme that we described above, could possibly be exploited for the design of specific inhibitors targeting the *M*. *tuberculosis* homologue.

### The catalytic site

As we were unable to obtain the crystal structure of MsPrsA in complex with either R5P or ATP/ADP, we modelled R5P and ATP/ADP in their respective binding sites by adopting a different approach for the two ligands, as detailed under the material and methods section. We could indeed model R5P by adopting a docking procedure that however proved unsuccessful when applied to ATP. We therefore attempted the modelling of ATP based on optimal superposition with the structure of *T*. *volcanium* PrsA, that we selected being the one that has been structurally investigated the most and reported in several liganded states [30]. As our structure is in the ligand-free form and therefore open conformation, we first attempted to modelling the adenine nucleotide based on the optimal superposition with the open form of *T*. *volcanium* PrsA in complex with either ADP or a non-hydrolysable ATP analog (PDB codes 3NAG and 3RLT, respectively). However, in both cases the ligand made severe clashes with MsPrsA residues that could not be resolved by energy minimization. On the contrary, no clashes were observed with the orientation that ADP-Mg^2+^ adopts in the closed conformation of *T*. *volcanium* PrsA (PDB code 3MBI) that was therefore selected for modelling an ADP-Mg^2+^ unit in the MsPrsA active site.

**Fig 9:**
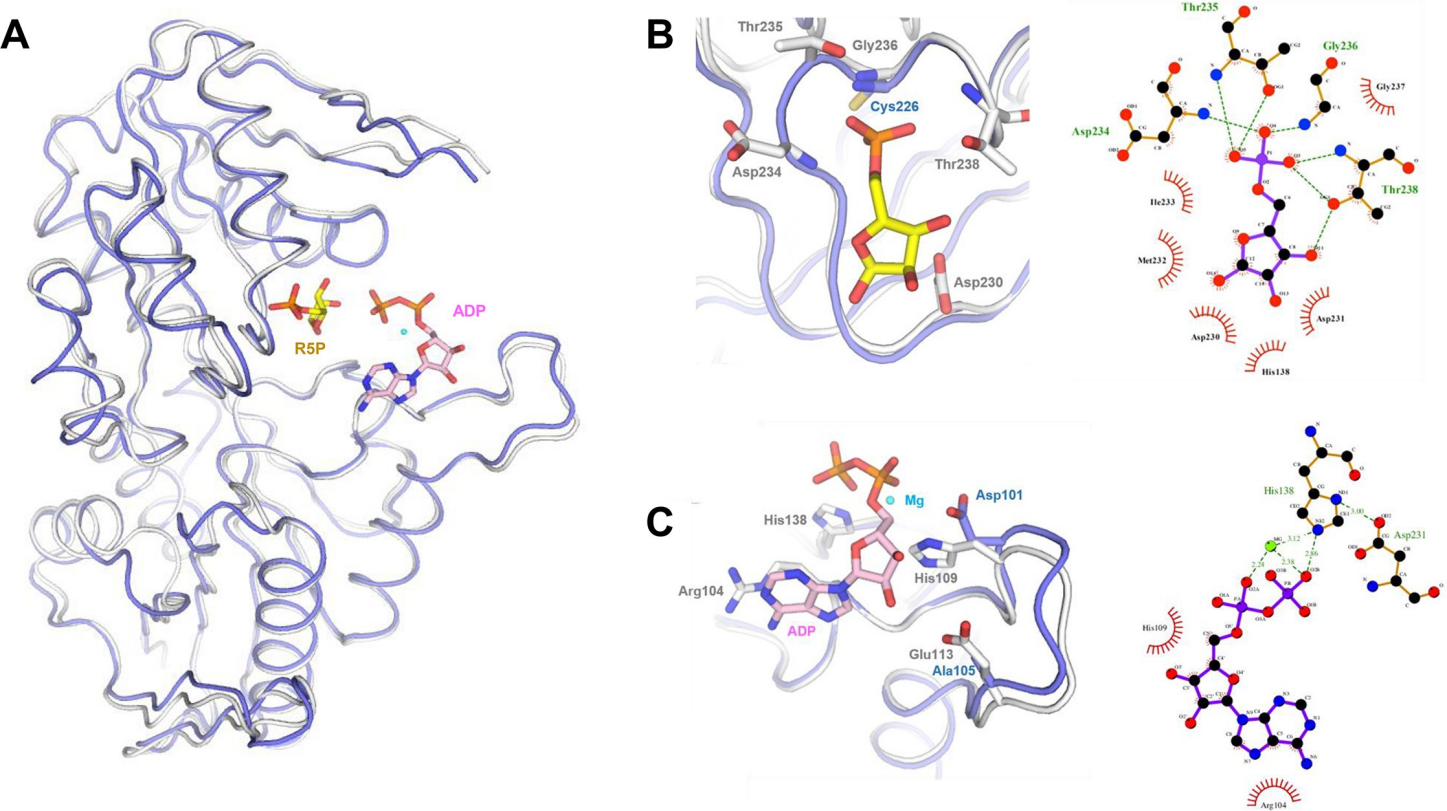
Modeled *M*. *smegmatis* R5P and ADP-Mg^2+^ binding sites superposed on the structure of hPrsA-I; hPrsA-1 and corresponding residues are shown in light blue and MsPrsA and corresponding residues are shown in white. (A) Ribbon representation of the overall structure of MsPrsA superimposed to hPrsa-I in which the R5P and ADP drawn as ball-and-sticks and represented with the same orientation reported in the respective model of the enzyme-ligand complex. (B) The R5P binding site on the left together with the schematic diagram of MsPrsA residues interacting with the ligand. (C) The ADP-Mg^2+^ binding site on the left together with the schematic diagram of MsPrsA residues interacting with the ligand.

The models of the *M*. *smegmatis* PrsA-complexes, i.e. the complex with R5P and with ADP-Mg^2+^, were analyzed with the main aim to identify potential differences in the protein residues involved in ligand binding between the mycobacterial and the human enzyme and were therefore superimposed on hPrsA-I (Fig 9A). As shown in Fig 9B, the modelled R5P molecule binds to conserved residues both in the mycobacteria and human enzymes (Asp230, Asp234, Thr235, Gly236, Thr238) except for Gly236 that in the *M*. *smegmatis* protein replaces Cys226 in the human enzyme. However, the interaction between the O4 of the phosphate group and Gly236 occurs through the amide carbonyl oxygen and therefore the difference appears as having an irrelevant effect on R5P binding. Similarly, modeled ADP is held in place by conserved interactions that involved major contributions by His138 that engages the nucleotide β-phosphate group, and Arg104 that contacts the adenine ring (Fig 9C). Remarkably, however, the two non-conserved residues His109 and Glu113 that, as described in the previous section occupies the structurally equivalent position in the human enzyme of an aspartic and alanine residues respectively, are both involved in ADP binding. Although the His to Asp replacement most likely does not significantly impact the ADP recognition and binding since both residues hydrogen bond to the ribose 3’ hydroxyl group, the other substitution opens a specific structural trait of the mycobacterial enzyme, being conserved in both *M*. *smegmatis* and *M*. *tuberculosis* PrsA (Fig 7). Indeed, the carboxylic group of Glu103 establishes a hydrogen bond with the ribose 2’ hydroxyl group (distance of 3.2 A) that, in the human enzyme, does not contact any protein residues and generates a more open ATP binding site.

## Discussion

It is well established that PRPP synthases are naturally prone to form multimers and that their oligomerisation is required for full enzymatic activity since both the catalytic site and allosteric regulatory site are shaped by conserved residues of neighbouring subunits [36,37,40].

MsPrsA is not an exception, and size-exclusion chromatography experiments reported here show an elution volume compatible with an hexameric quaternary structure, consistent with what observed for other Class I PRPP synthase [24,41], and therefore hinting at the hexamer as the physiological oligomeric state required for catalysis and enzymatic function; a fact that is further supported by the crystal structure that shows the MsPrsA assembled into an hexamer.

The enzyme characterisation of MsPrsA reveals that the protein activity is highly regulated by phosphate and Mg^2+^ ions. We observed that Pi affects the enzyme activity by increasing the catalytic rate peaking at 50 mM Pi concentration while at higher concentrations the ion exerts inhibitory effects, showing the typical behaviour of Class I PRPP synthases [24,42,43].

Further analysis investigating substrate specificity and catalytic activity towards R5P and ATP showed a classical Michaelis-Menten hyperbolic kinetics in the case of R5P and a sigmoidal behaviour for ATP with a calculated Hill coefficient of 1.7 that indicates a ATP cooperative binding as also observed in MtPrsA [13].

MsPrsA highly favours ATP as the diphosphate donor, and appears almost inactive in the presence of either ADP or GDP. These two features further connote MsPrsA as a member of Class I PRPP synthases. The MsPrsA half-maximal inhibition concentration values for GDP and ADP (58 μM and 79 μM, respectively) differ significantly from the ones reported for both *M*. *tuberculosis* and human orthologs [43], indicating a more strict regulation exerted by these diphosphate nucleosides on the *M*. *smegmatis* enzyme.

Class I PRPP synthases are typically characterised by Mg^2+^ and phosphate requirement for their enzymatic activity, allosteric inhibition by ADP and other nucleotides, and by a functional hexameric structure. Overall, the biochemical, biophysical and structural data reported here identify MsPrsA as a Class I PRPP synthase and describe MsPrsA as a highly regulated enzyme that can adjust its activity to the many environmental challenges encountered during the bacterial lifespan.

Not surprisingly, the crystal structure of MsPrsA shows an overall enzyme architecture consistent with other Class I PRPP synthases, with conserved fold and oligomeric state. As the crystal structure we determined is the one of the ligand-free form, we analysed the R5P and ATP binding mode by modelling the ligands in their respective binding sites by optimal structural superposition and energy minimization. While the R5P binding site does not reveal any significant peculiarity in MsPrsA and is confirmed as highly structurally conserved, significant differences are observed in the case of the ATP binding compared to the human ortholog. Although the only significant difference observed in the R5P binding site, where the Gly236 occupies the structurally equivalent position of Cys226 in the human enzyme, does not seem to directly impact ligand binding, it could be of relevance for the design of specific inhibitors as it creates a more open R5P binding pocket in the mycobacterial enzyme in comparison the human counterpart. More significant instead are the two differences observed in the ATP binding pocket where the recognition of the ribose portion of the nucleotide is completed in the mycobacterial enzyme with a Glutamic residue that replaces an Alanine in the equivalent position in the human enzyme (Glu113 vs Ala105). The presence of a glutamate side chain in the MsPrsA opens the possibility to design an inhibitor containing a moiety capable of interacting with Glu113 through a hydrogen bond and/or an electrostatic interaction; such a possibility is simply hampered in the human enzyme where an Ala residue is present. Moreover, the long and flexible glutamate side chain exists in different conformers, therefore allowing the accommodation of a ligand/inhibitor able to establish those stabilising interactions. Overall this could lead to the design of a potent and specific mycobacterial enzyme inhibitor. Taken together, these observations suggest that the issue of inhibitor specificity could be challenged and specific *M*. *tuberculosis* PrsA inhibitors possibly obtained.

The difficult, expensive and demanding handling of *M*. *tuberculosis* in a laboratory setting leads the scientific community to look at valid and less problematical alternative for the study of *M*. *tuberculosis* pathogenesis and biology. In this context, *M*. *smegmatis* represents a valid surrogate model for the study of *M*. *tuberculosis* and its biology due to its convenient handling, fast doubling times, ease of genetic manipulation and close genetic affinity towards its pathogenic counterpart [15,44–46], although doubts and skepticisms still exist [47]. Although not perfect, the study of surrogate model of tuberculosis provides a “better than nothing” approach that must not be underestimated in a field where all the information available can contribute to the overall better knowledge of a highly mortal, global threat such as tuberculosis.
